# Low-Contrast High-Pass Visual Acuity Might Help to Detect Glaucoma Damage: A Structure-Function Analysis

**DOI:** 10.3389/fmed.2021.680823

**Published:** 2021-05-14

**Authors:** Yun Wen, Zidong Chen, Chengguo Zuo, Yangfan Yang, Jiangang Xu, Yang Kong, Hui Cheng, Minbin Yu

**Affiliations:** ^1^State Key Laboratory of Ophthalmology, Zhongshan Ophthalmic Center, Sun Yat-sen University, Guangzhou, China; ^2^School of Electronics and Communication Engineering, Sun Yat-sen University, Shenzhen, China; ^3^School of Computer Science and Engineering, Sun Yat-sen University, Guangzhou, China

**Keywords:** low-contrast visual acuity, high-pass optotypes, glaucoma, macular damage, optical coherence tomography

## Abstract

**Purpose:** The conventional visual acuity (VA) test is not sensitive enough to detect glaucoma macular damage. We aimed to investigate whether VA measurements using low-contrast high-pass optotypes are more sensitive to macular dysfunction in glaucoma and to find the potential structural basis of this difference.

**Methods:** A total of 147 subjects were recruited, including 118 patients with glaucoma (mean age: 46.08 ± 14.64 years) and 29 healthy controls (mean age: 39.83 ± 9.81 years). For each participant, monocular best-corrected VA was measured using a conventional chart and six high-pass charts at 100, 50, 10, 5, 2.5, and 1.25% contrast levels, respectively. The macular retinal thickness and circumpapillary retinal nerve fiber layer (cpRNFL) thickness of all the glaucoma patients were obtained by spectral-domain optical coherence tomography (SD-OCT).

**Results:** Compared with healthy subjects, glaucoma patients with normal vision demonstrated worse VAs in high-pass acuity measurements (0.22–0.93 vs. 0.28–1.08, *p* < 0.05). Receiver operating characteristic curve (ROC) showed that 1.25% low-contrast high-pass VA was optimal for discriminating between the controls and glaucoma patients (AUC: 0.918, *p* < 0.001; sensitivity: 77.33%; specificity: 96.55%). Compared with conventional VA, 1.25% high-pass VA correlated better with nasal-side macular retinal ganglion cell (RGC)-related parameters (*r* = −0.419 to −0.446 vs. *r* = −0.538 to −0.582; Fisher's Z transformation, *p*_z_ < 0.05). There was no difference in the strength of correlations between the VAs measured using different charts and cpRNFL thickness (Fisher's Z transformation; *p*_z_ > 0.05).

**Conclusions:** VA measurement taken with low-contrast (1.25%) high-pass acuity chart is more sensitive in detecting central visual loss in glaucoma than that taken with the conventional chart. Macular RGC damage appears to be associated with low-contrast (1.25%) high-pass visual loss in glaucomatous eyes.

## Introduction

Glaucoma is the most frequent cause of irreversible blindness and visual impairment worldwide. It has been projected to affect around 112 million people by 2040 (1). The common features of glaucoma are loss of RGCs, thinning of the cpRNFL, cupping of the optic disc and visual field (VF) defects (1, 2). Glaucoma has traditionally been regarded as an insidious disease that features progressive loss of peripheral vision and sparing of macular vision until late in the process of the disease (3). This perception was based on the VA test, the most common clinical measurement used to assess macular visual function (3). This subjective and rough method evaluates only the resolution ability of the eye at a fixed high contrast. However, resolving power is only one aspect of the very complex central visual perception pathway. Many glaucoma patients have complaints regarding central vision despite normal VA (4, 5).

Accumulating evidence shows that macular involvement occurs earlier in glaucomatous eyes than once thought (6–8). Studies investigating reading speed (9) and facial recognition (10) have reported that the macular vision of glaucomatous eyes is significantly compromised. Other studies have confirmed that spatial contrast sensitivity (CS) declines in glaucoma patients, even those with normal VA, specifically at the high spatial frequency end (11). However, it is important to have a functional test that is sensitive to glaucoma macular damage that can be conveniently conducted in busy clinical practices and easily understood by patients.

There are two distinct visual thresholds regarding the conventional black-on-white letters, specifically the detection threshold and the recognition threshold (12). Significant differences in the low spatial frequency content of the conventional letters make them more identifiable at a much greater distance than the actual resolution required (12). Thus, this discrepancy between detection and resolution thresholds can help to achieve superior levels of VA. Howland et al. have devised a special type of optotype called high-pass spatial frequency letters (13). The special design of these stimuli make the low spatial frequencies removed and appear as letters with a black core and a white outline (or vice versa) with their mean luminance equal to that of the gray background. In healthy eyes, the detection threshold almost coincides with the resolution threshold under foveal viewing.

VA measured by high-pass filtered optotypes demonstrated higher sensitivity to neural limitations of age-related macular degeneration (AMD) damage than conventional black-on-white letters (14). However, it is reasonable to speculate that undersampling as a result of RGC loss around the fovea in glaucomatous eyes may cause discrepancies between detection and resolution thresholds, resulting in acuity loss for high-pass filtered letters.

Previous studies on patients with multiple sclerosis and optic neuritis confirmed that acuity charts with a set of variable contrasts provide qualitatively similar diagnostic information to that provided by the sinusoidal gratings of different contrast and different spatial frequencies in CS tests (15, 16). Patients with glaucoma also exhibited visual loss in the low-contrast acuity test (17). However, VA tests using high-pass filtered optotype settings at various contrasts have not yet been performed in glaucoma patients. Do these charts offer simple and more sensitive ways of detecting macular dysfunction in glaucoma? To answer this question, we compared the test results of various contrast high-pass VA charts between glaucoma patients and healthy participants and explored the structure-function relationships between OCT parameters and VA results in glaucoma patients.

## Materials and Methods

### Patients

This study was performed according to the tenets of the Declaration of Helsinki and was approved by the ethics committee of the Zhongshan Ophthalmic Center (NO.2019KYPJ115). Written informed consent was obtained from all participants prior to the experiment. The subjects were recruited from the Glaucoma Clinic at Zhongshan Ophthalmic Center.

The patients with glaucoma in the current study met the following criteria: (1) a diagnosis of primary open-angle glaucoma (POAG) or normal tension glaucoma (NTG) in one or both eyes as determined by at least two glaucoma specialists (2); In the current study, glaucoma was diagnosed based on characteristic optic nerve damage on stereoscopic imaging, cpRNFL thinning on SD-OCT, open anterior chamber angles on gonioscopy and absence of other known explanations of progressive glaucomatous optic nerve change. (2) A best-corrected visual acuity (BCVA) better than or equal to 0.20 logMAR (Early Treatment Diabetic Retinopathy Study logMAR chart, ETDRS chart) in the eye; (3) spherical equivalents between −6.0 diopters (D) and +3.00 D and cylinder correction within ±3D; (4) no N2 or worse nuclear sclerotic cataract graded by the Lens Opacities Classification System III criteria (18) or any posterior subcapsular or cortical lenticular changes in the lens; (5) no severe dry eye or other ophthalmic surface diseases; and (6) no ocular or systemic disease that could affect the optic nerve, macula, or VF results. Finally, a total of 147 subjects were recruited, including 118 patients with glaucoma (110 patients with POAG and 8 patients with NTG; mean age 46.08 ± 14.64 years) and 29 age-similar healthy controls (mean age 39.83 ± 9.81 years) with BCVA equal to or better than 0.00 logMAR on ETDRS acuity chart.

To determine the best-corrected refractive correction and the BCVA of each enrolled eye, a cycloplegic refraction was done on each participant within a week before the experiment. Objective refraction was measured by autorefraction (NIDEK ARK-1) first. Then subjective refinements to achieve the best VA and optimum optical correction were performed using a phoropter (NIDEK RT-5100). The contemplated prescription was then used in a trial frame for monocular VA measurements performed with the ETDRS illuminator cabinets (Precision Vision, Inc., USA; illuminance, 160 cd/m^2^) at a distance of 4 m.

### VA and Contrast Testing

#### Apparatus

High-pass VA was measured using specially designed electronic charts (e-charts) generated by MATLAB (MathWorks, Inc., Natick, MA) with the Psychophysics Toolbox for Windows 10, administered on a laptop computer. The e-charts were displayed on a liquid crystal display monitor (DELL, P2415Q, 23.8 inches, resolution: 3,840 × 2,160, refresh rate: 60 Hz). Luminance of the display monitor was made linear after gamma correction using a TES-1330A Digital Light Meter (TES Electrical Electronic Corp., Taipei, Taiwan). The values of the properties for the display of stimuli in the current study were specified in pixels. The brightness of the screen and the surround luminance was kept consistent. The screen brightness was set at 100%. The room lights were turned off on the side of the screen, ensuring a stable ambient illumination of 8 lux, while the lights on the side of the subjects remained on, providing a luminance of 160 cd/m^2^. Participants were seated on a chair with a vertical back 4 meters away from the front of the screen.

#### Stimuli

**Figure 1:**
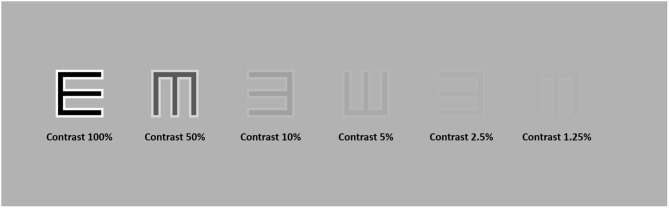
High-pass letter “E” at six different contrast levels, namely, 100, 50, 10, 5, 2.5, and 1.25%. The contrast was defined as Michelson Contrast.

The e-charts employed the same layout as the current standard ETDRS chart, with optotype sizes ranging from 58.18 to 2.92 mm, providing a test range from 1.0 logMAR to −0.3 logMAR at a 4 m distance. We used the 5 × 5 letter “E” with a lighter edge (luminance: 228 cd/m^2^) and a darker core (luminance: 3 cd/m^2^), which formed a constant ratio of 1:2:1 (edge: core: edge), as the optotype design (high-pass design). The mean luminance of the strokes was consistent with the luminance of the gray background (luminance: 112 cd/m^2^). The contrast was defined as Michelson contrast (see Figure 1). During the test, the visual chart went line by line on the center of the screen. In each line, there were five optotypes with interval spaces as one letter width. The high-pass “E” was presented randomly in four directions: left, right, up, and down.

#### Test Procedure

The best-corrected refractive correction was used for each participant before the tests. For each VA test in the current study, participants were required to identify every optotype in each row by a forced choice procedure. They were instructed to identify the orientations of the high-pass “E”s by pressing direction buttons on a Bluetooth keyboard. Testing time was not restricted. Once the participants were unsure of an optotype, they were encouraged to guess. The tests automatically stopped when four or more errors occurred in a row. Then, the final VA score was calculated using the method described by Ferris et al. (19). When a participant could not correctly read at least 4 letters of the top row at 4 m, the test distance was reduced to 1 m. In this case, only the top 6 rows were required, and a +0.75DS was added to the prescription in the trial frame as the refractive compensation for the distance reduction. In the current study, VA was scored as logMAR values on a by-letter basis. High-pass VAs were measured at contrast settings of 100, 50, 10, 5, 2.5, and 1.25%. Participants were allowed to take a 5-min break between tests to minimize the effects of fatigue.

For comparison, the conventional VA test was also conducted using the same set of apparatuses. The black-on-white VA chart followed the design of the current standard ETDRS chart. The test procedure and scoring rules were all in accordance with the high-pass VA tests.

### SD-OCT Scan

**Figure 2:**
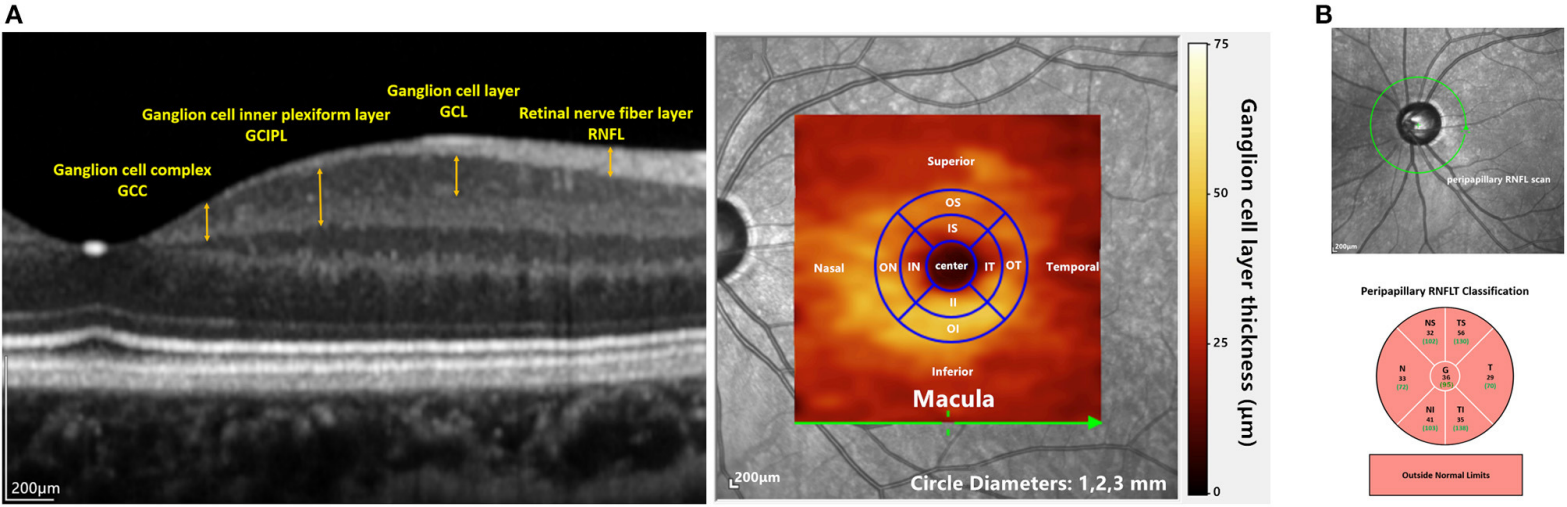
OCT imaging in the current study: **(A)** the left image shows macular layers that were used for analyzing the structure-function relationships in this study. The right panel shows the overlay of the GCL thickness heat map on a fundus image. The inner and outer annuli were divided into four quadrants (IS, IT, II, IN and OS, OT, OI, ON) according to the ETDRS map. **(B)** the upper image shows the cpRNFL scan. The lower picture shows the sectoral cpRNFL thicknesses classification, specifically the global average of the circle scan (G) and N, NS, TS, T, TI, and NI quadrants.

Macular retinal layer thickness of each enrolled eye was acquired by a well-trained ophthalmic photographer using SD-OCT (Spectralis, Heidelberg Engineering GmbH, Heidelberg, Germany). The macular images were generated using the “Dense” protocol in high-resolution volume scan mode with an automatic real-time mean value of 15. The imaging covered a 6 × 6 mm area of the macula centered on the fovea. The thickness of each layer was segmented and calculated by the automatic segmentation algorithms of the built-in software (Version 6.3.4). Scans were acquired with 49 B-scans consisting of 1,024 A-scans. All scans were reviewed, and any scan with a quality score <20 dB or segmentation error was excluded from analysis. The ganglion cell complex (GCC) layer thickness was the sum of the thickness of the retinal nerve fiber layer (RNFL), ganglion cell layer (GCL), and inner plexiform layer (IPL); the ganglion cell inner plexiform layer (GCIPL) thickness was the combination of the GCL and IPL (see Figure 2A). The average retinal thickness and retinal volume were divided into nine subfields according to the ETDRS grid, specifically a central subfield (diameter 1 mm), the inner ring (radius 0.5 mm and radius 2 mm) and the outer ring (radius 3 mm). The inner ring and outer ring were automatically divided into four quadrants by in-build software: superior, nasal, inferior and temporal (see Figure 2A).

The cpRNFL protocol was also conducted, in which 3.4-mm-diameter circle scans were acquired. Sectoral cpRNFL thicknesses, specifically the global average of the circle scan (G), nasal (N), superonasal (NS), superotemporal (TS), temporal (T), inferotemporal (TI), and inferonasal (NI) quadrants, provided by the built-in software were read (see Figure 2B).

### Data Analysis

We first compared the VA data between glaucomatous eyes with normal vision (*N* = 75) and healthy controls (*N* = 29). Here, normal vision was defined as BCVA equal to or better than 0.00 logMAR on the ETDRS VA chart. The normality of the data was checked using the Shapiro-Wilk test. Bland-Altman plots (20) were used to display the comparison results for the different charts in the glaucoma group and healthy control group. Regression analysis was used to quantify any potential proportional bias. The discrimination performance of the VA tests in glaucoma damage was assessed by ROC curve analysis. Areas under the curves (AUCs) were calculated to compare the discriminative value of each VA test. The optimal cutoff value was obtained according to Youden index analysis as the points with the best sensitivity-specificity balance.

Then, the correlations between multiple OCT parameters and VA results were evaluated by Pearson's partial correlation analysis after adjusting for age and spherical equivalent (SE) in all glaucoma participants (*N* = 118). Then Fisher's Z transformation was conducted for comparisons of the correlations.

Statistical analysis was conducted using SPSS for Windows (version 20.0; SPSS, Inc., Chicago, IL, USA), the GraphPad Prism statistical analysis package (version 7.00; GraphPad Software, Inc., La Jolla, California, USA) and MedCalc statistical software (version 19.0.4; MedCalc Software Ltd, Ostend, Belgium).

## Results

### The Test-Retest Reliability of the Conventional Chart and the High-Pass Charts

**Figure 3:**
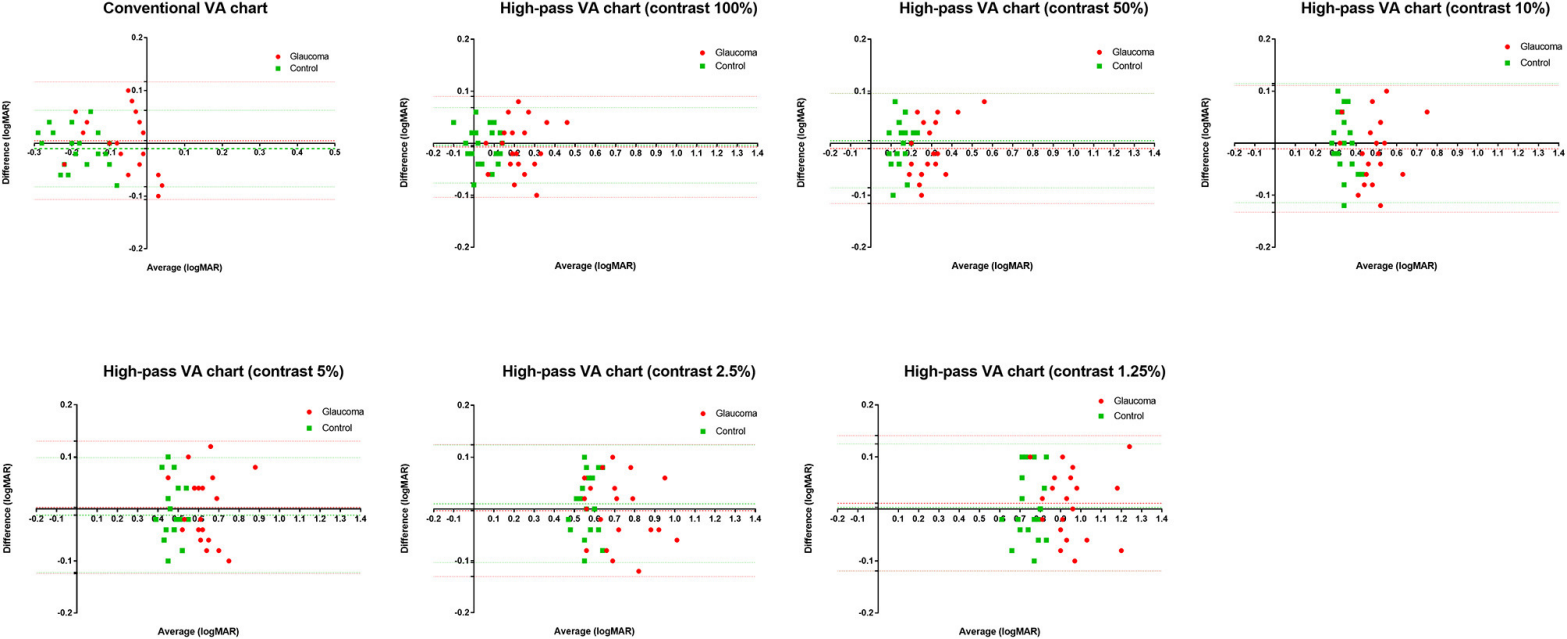
Bland-Altman plots for test-retest measurements for the conventional chart (1st test vs. 2nd test) and the high-pass charts (1st test vs. 2nd test) with data for the 20 healthy controls plotted in green (squares) and the 20 glaucoma patients in red (dots). The mean difference of the two tests and 95% Limits of Agreements are also shown in the plots (dotted lines).

In this part, 20 glaucoma patients with BCVA equal to or better than 0.00 logMAR on ETDRS chart and 20 healthy controls underwent VA tests under the same conditions at two different points in time. The test-retest reliability was analyzed using Bland-Altman plots (see Figure 3).

The mean difference between the two tests (the 1st test and the 2nd test) and the 95% limits of agreement (LoA) of each VA chart were calculated separately (see Table 1).

**Table 1:** Test-retest reliability of the conventional chart and the novel high-pass charts.

	**Mean difference of 1st and 2nd test**	**TRV, 95% Limits of agreement**
**Conventional chart**
Healthy control	−0.010 ± 0.037	−0.083 to 0.063
Glaucoma	0.005 ± 0.057	−0.107 to 0.117
**High-pass chart (contrast 100%)**
Healthy control	−0.004 ± 0.037	−0.077 to 0.069
Glaucoma	−0.007 ± 0.050	−0.104 to 0.090
**High-pass chart (contrast 50%)**
Healthy control	0.005 ± 0.046	−0.086 to 0.096
Glaucoma	−0.010 ± 0.054	−0.116 to 0.096
**High-pass chart (contrast 10%)**
Healthy control	0.000 ± 0.058	−0.114 to 0.115
Glaucoma	−0.011 ± 0.062	−0.133 to 0.111
**High-pass chart (contrast 5%)**
Healthy control	−0.012 ± 0.056	−0.122 to 0.098
Glaucoma	0.003 ± 0.065	−0.123 to 0.130
**High-pass chart (contrast 2.5%)**
Healthy control	0.010 ± 0.058	−0.103 to 0.123
Glaucoma	−0.003 ± 0.065	−0.130 to 0.124
**High-pass chart (contrast 1.25%)**
Healthy control	0.003 ± 0.062	−0.119 to 0.125
Glaucoma	0.011 ± 0.066	−0.119 to 0.141

*TRV, Test-retest variability*.

### Visual Acuity Measured Using Conventional Chart and High-Pass Charts in Glaucomatous Eyes With Normal Vision

**Figure 4:**
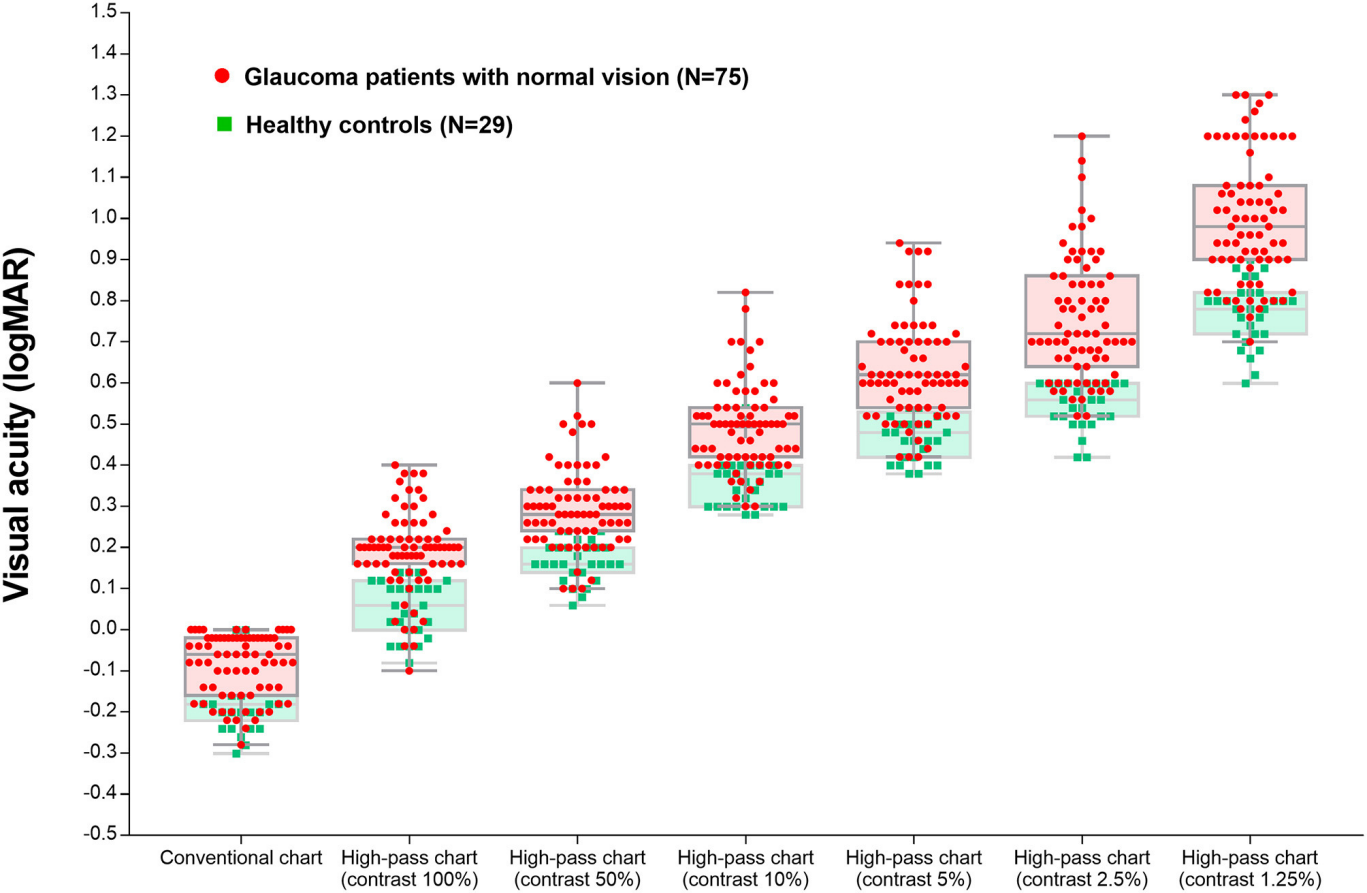
Visual acuity measured using the conventional chart and high-pass charts at 100, 50, 10, 5, 2.5, and 1.25% contrast levels among glaucoma patients with normal vision and the healthy controls.

In this section, we tried to determine whether low-contrast high-pass charts are more sensitive for detecting central visual dysfunction in glaucomatous eyes. Thus, we compared the VA data between glaucoma patients with normal vision and healthy controls. A total of 75 glaucomatous eyes with BCVA equal to or better than 0.00 logMAR and 29 healthy eyes were included in the analysis. The characteristics of the participants are summarized in Table 2. VAs measured using conventional chart and high-pass charts at 100, 50, 10, 5, 2.5, and 1.25% contrast levels among the two group of participants are shown in Figure 4.

**Table 2:** Characteristics of the glaucoma patients with normal vision and the healthy controls.

	**Glaucoma patients with normal vision (*N* = 75)**	**Healthy controls (*N* = 29)**	***P*-value**
Age, years	43.19 ± 13.98	39.83 ± 9.81	0.239
Gender, F/M	32/43	16/13	0.251
Spherical equivalents, diopters	−2.02 ± 2.59	−2.24 ± 2.28	0.683
MD of 30-2 VF, dB	−10.48 ± 7.82	−1.74 ± 1.30	<0.001
BCVA, logMAR	−0.09 ± 0.08	−0.17 ± 0.07	<0.001

*P-value was obtained from independent sample t-test, except gender data was compared using Chi-squared Test*.

*F/M, female/male; MD, mean deviation; VF, visual field; BCVA, best-corrected visual acuity; logMAR, the logarithm of the minimum angle of resolution*.

The differences in VAs measured using conventional chart and each high-pass chart in the glaucoma group and healthy control group are summarized in Table 3. The difference between conventional VA and high-pass VA at any one of the contrast levels was significantly different between the two groups of participants (*p*_100%_ = 0.004, *p*_50%_ = 0.014, *p*_10%_ = 0.007, *p*_5%_ < 0.0001, *p*_2.5%_ < 0.0001, *p*_1.25%_ < 0.0001).

**Table 3:** Differences in visual acuities measured using conventional chart and high-pass charts in glaucoma patients with normal vision (*N* = 75) and the healthy controls (*N* = 29).

**Reference: Conventional VA**	**Differences, Mean** **±** **SD (95% CI)**		***P*-value (two-tailed)**
	**Healthy controls**	**Glaucoma patients**	
	**(*N* = 29)**	**(*N* = 75)**	
High-pass VA (contrast 100%)	0.22 ± 0.08 (0.19–0.26)	0.28 ± 0.07 (0.26–0.29)	0.004
High-pass VA (contrast 50%)	0.33 ± 0.06 (0.31–0.36)	0.38 ± 0.08 (0.36–0.39)	0.014
High-pass VA (contrast 10%)	0.53 ± 0.05 (0.50–0.55)	0.57 ± 0.08 (0.55–0.59)	0.007
High-pass VA (contrast 5%)	0.64 ± 0.06 (0.62–0.67)	0.71 ± 0.09 (0.69–0.74)	<0.0001
High-pass VA (contrast 2.5%)	0.73 ± 0.05 (0.71–0.75)	0.84 ± 0.12 (0.81–0.86)	<0.0001
High-pass VA (contrast 1.25%)	0.93 ± 0.07 (0.90–0.96)	1.08 ± 0.13 (1.05–1.11)	<0.0001

*P-value was obtained from independent sample t-test; VA, visual acuity*.

**Figure 5:**
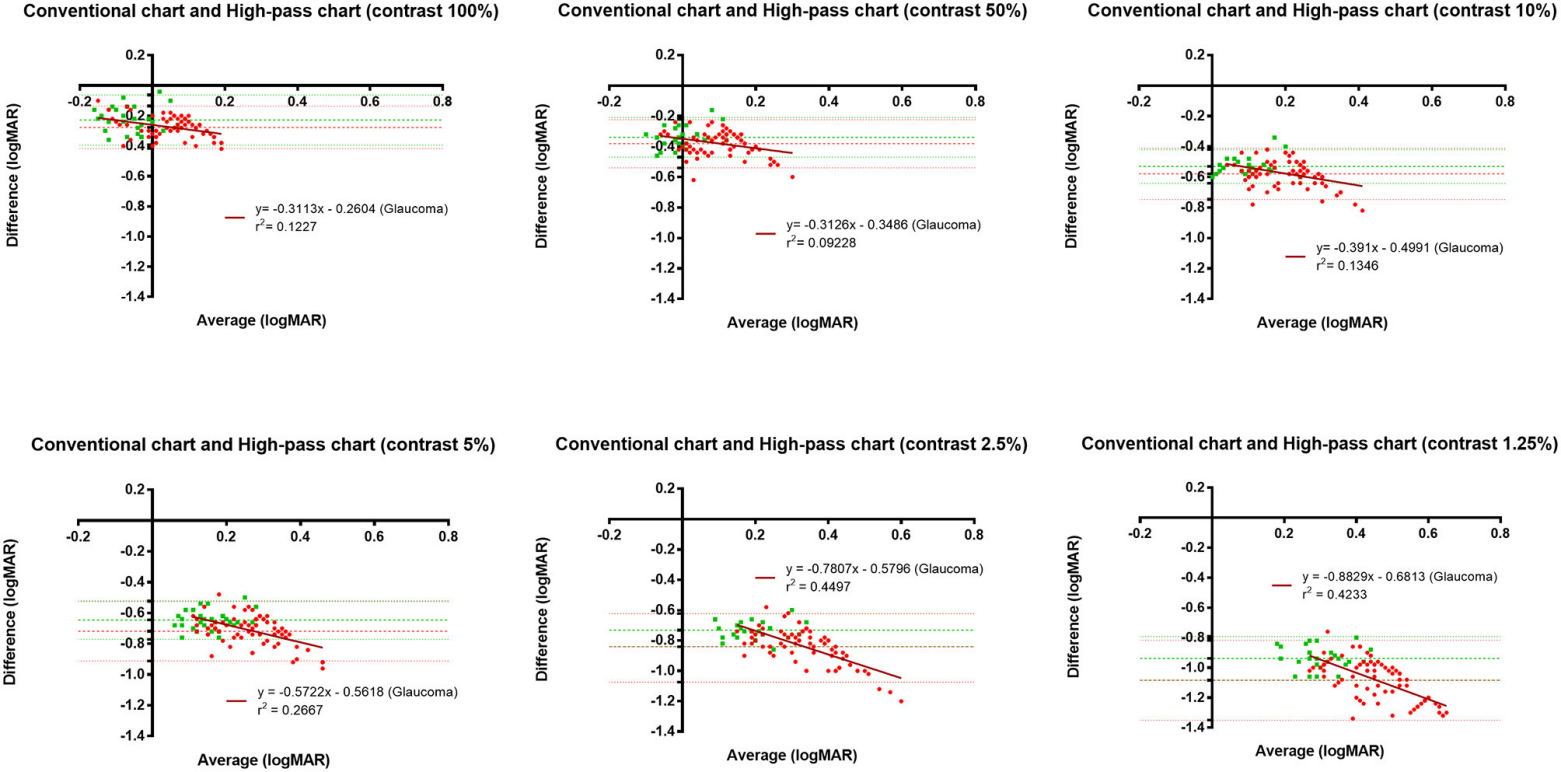
Bland-Altman plots displaying the differences in VAs measured using the conventional chart and high-pass charts in glaucomatous eyes with normal vison (*n* = 75) and healthy controls (*n* = 29). The red dots represent data from glaucoma patients, whereas the green squares represent data for healthy controls. The horizontal lines represent the bias of the tests and 95% limits of agreement. The dark red lines represent the best linear fit to the data from glaucoma patients.

Figure 5 displays the difference in VAs measured using conventional chart and high-pass charts between the glaucoma group with normal vision (in red) and the healthy controls (in green). We can see that there was a greater level of disagreement at the worse acuity end on the pattern form by the data from glaucoma patients. Regression analysis confirmed that these proportional biases were statistically significant (*p*_100%_ = 0.0021, *p*_50%_ = 0.0081, *p*_10%_ = 0.0012, *p*_5%_ < 0.0001, *p*_2.5%_ < 0.0001, *p*_1.25%_ < 0.0001). As the contrast decreased, the difference between VAs became larger. At the 1.25% contrast level, the slope of the regression line reached −0.88. When we looked at the pattern formed by the data from healthy controls, the difference was relatively constant throughout, and the regression analysis showed no statistical significance (*p*_100%_ = 0.9992, *p*_50%_ = 0.0585, *p*_10%_ = 0.1702, *p*_5%_ = 0.2828, *p*_2.5%_ = 0.8431, *p*_1.25%_ = 0.8294).

**Figure 6:**
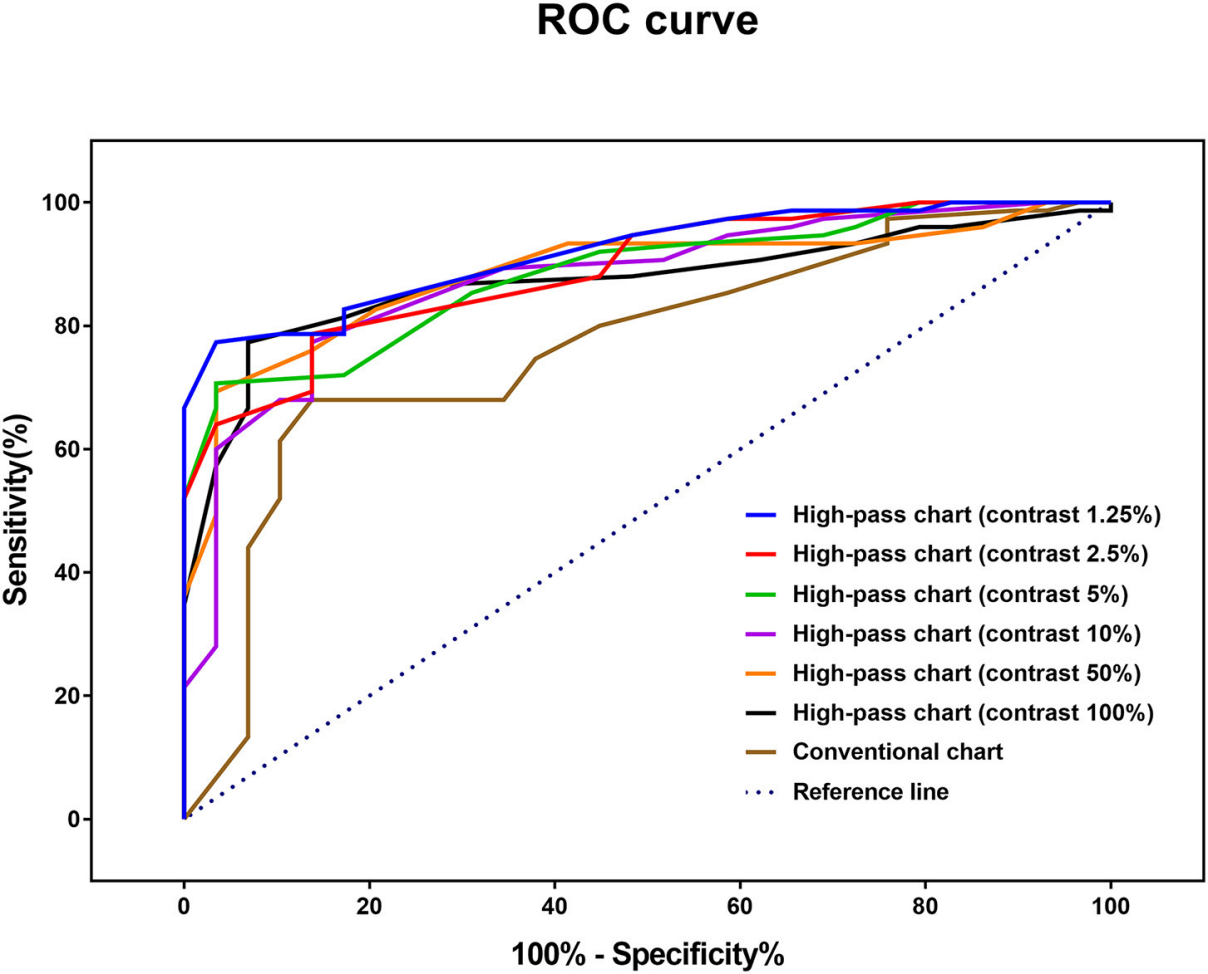
Receiver operating characteristic (ROC) curve for each VA test to detect glaucoma damage.

ROC curve analysis was performed to determine the optimal method for discriminating glaucomatous eyes from healthy eyes (Figure 6). The AUCs of the high-pass charts were larger than that of the conventional chart, with the highest figure peaking at 0.918 (95% CI: 0.847–0.963), showing at 1.25% contrast level (Figure 6). In addition, the optimal cutoff point of each VA test was obtained from the Youden index with the best sensitivity-specificity balance (Table 4).

**Table 4:** Receiver operating characteristic curve analysis of visual acuities between glaucoma patients with normal vison (*N* = 75) and healthy controls (*N* = 29).

	**AUC (95% CI)**	***P*-value**	**Cutoff value**	**Sensitivity**	**Specificity**	**Youden index**
**Conventional chart**	0.768 (0.675–0.845)	<0.0001	−0.12	68.00	86.21	0.5421
**High-pass chart**
Contrast 100%	0.872 (0.792–0.929)	<0.0001	0.14	77.33	93.10	0.7044
Contrast 50%	0.883 (0.806– 0.938)	<0.0001	0.24	69.33	96.55	0.6589
Contrast 10%	0.874 (0.794–0.931)	<0.0001	0.40	77.33	86.21	0.6354
Contrast 5%	0.883 (0.805–0.937)	<0.0001	0.56	70.67	96.55	0.6722
Contrast 2.5%	0.889 (0.812–0.942)	<0.0001	0.60	78.67	86.21	0.6487
Contrast 1.25%	0.918 (0.847–0.963)	<0.0001	0.88	77.33	96.55	0.7389

*AUC, area under the ROC curve; CI, confidence interval*.

### Structure-Function Relationship Between Visual Acuity and Retinal Thickness Measured by SD-OCT in Glaucoma Patients

**Figure 7:**
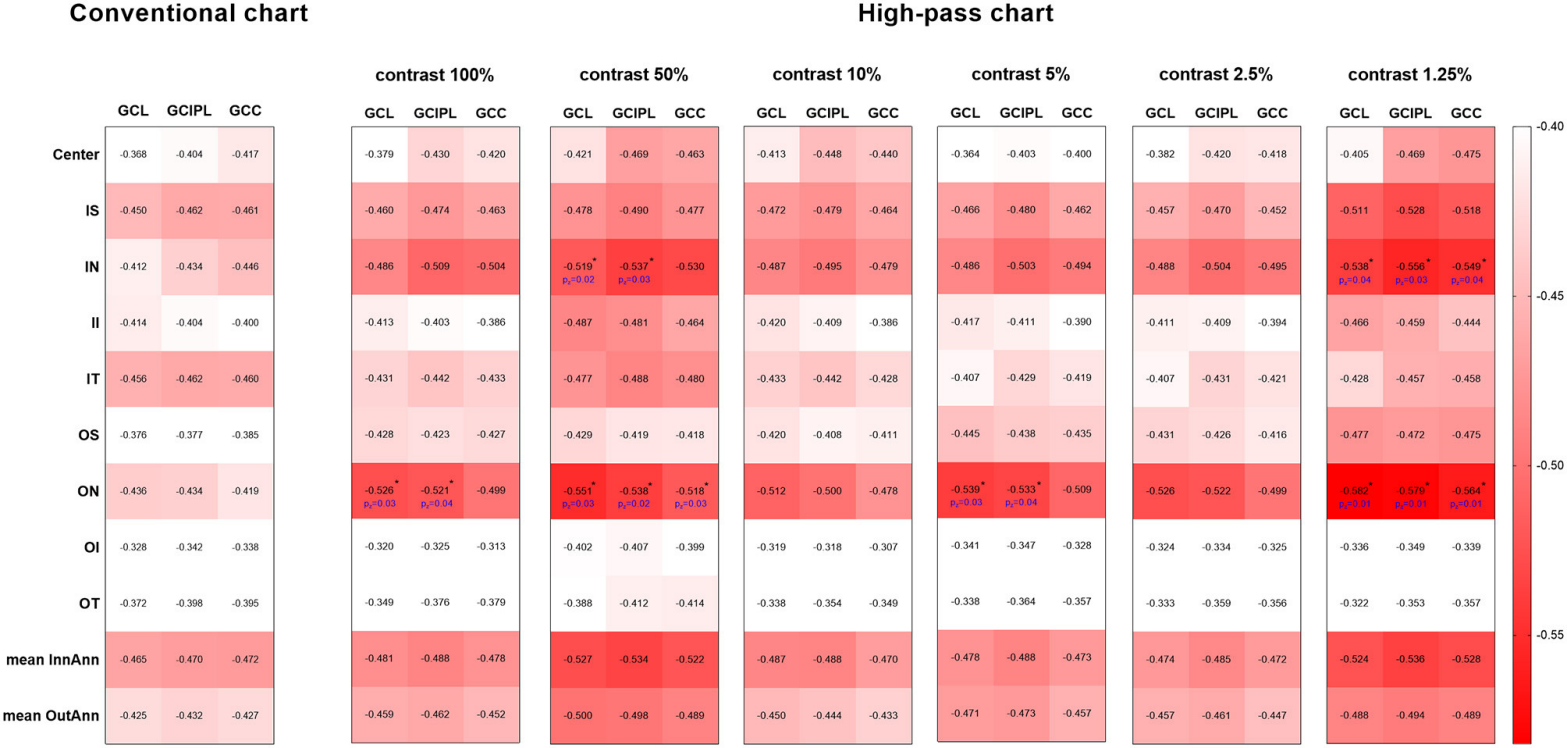
Heat map demonstrates the correlations between each parameter of the macular scan and VA data (Pearson's partial correlation analysis). The red-scale strength range of the correlation coefficients are shown on the right side of the plots. The correlations shown above were all statistically significant at the level of 0.01. The values marked with asterisks are statistically significant at *p* < 0.05 level (one-tailed) in Fisher's Z transformation and represent stronger structure-function correlations than those between structure parameters and conventional VA. GCL, ganglion cell layer; GCIPL, ganglion cell inner plexiform layer; GCC, ganglion cell complex; IS, inner superior; IN, inner nasal; II, inner inferior; IT, inner temporal; OS, outer superior; ON, outer nasal; OI, outer inferior; OT, outer temporal; InnAnn, inner annulus; OutAnn, outer annulus.

In this section, data from a total of 118 glaucomatous eyes were analyzed. The BCVA was −0.02 ± 0.11 logMAR and the mean deviation (MD) of 30-2 VF was −13.05 ± 8.60 dB. The correlations between OCT parameters and the VAs were examined by Pearson's partial correlation adjusted for age and SE. There were significant correlations between VAs and the overall RGC-related parameters (GCL, GCIPL, and GCC) of macular scans (Figure 7). Among all the VAs, high-pass VA with the 1.25% contrast setting showed the higher correlations with most of the macular scan parameters, especially RGC-related parameters of the nasal (−0.538 to −0.582, *p* < 0.001) and superior subfields (*r* = −0.472 to −0.528, *p* < 0.001). Fisher's Z transformation confirmed that high-pass VA at 100, 50, and 5% contrast level demonstrated slightly stronger correlations with some of the nasal-side parameters when compared with that of conventional VA (one-tailed *p*_z_, < 0.05), and 1.25% low-contrast high-pass VA demonstrated stronger structure-function relationships with all of the nasal-side RGC-related parameters (Fisher's Z transformation; one-tailed *p*_z_, < 0.05).

The correlations between cpRNFL parameters and the VAs are summarized in Table 5. Temporal-side RNFL thickness had the strongest correlations with the low-contrast high-pass VAs at 10, 5, 2.5, and 1.25% contrast settings (*r* = −0.367 to −0.439, *p*_r_ < 0.05). The conventional VA and high-pass VAs at 100 and 50% contrast settings showed slightly better correlations with the global average cpRNFL thickness (*r* = −0.347, −0.403, and −0.399, respectively; *p*_r_ < 0.05). However, Fisher's Z transformation confirmed that there is no difference in the strength of correlations between VAs measured using different charts and cpRNFL thickness (Fisher's Z transformation; two-tailed *p*_z_ > 0.05).

**Table 5:** Correlations between cpRNFL thickness and visual acuity results.

	**Conventional chart, r**	**High-pass charts**, ***r***					
		**Contrast 100%**	**Contrast 50%**	**Contrast 10%**	**Contrast 5%**	**Contrast 2.5%**	**Contrast 1.25%**
TS	−0.320	−0.360	−0.338	−0.313	−0.330	−0.315	−0.310
T	−0.340	−0.378	−0.363	−0.367	−0.397	−0.367	−0.439
TI	−0.259	−0.267	−0.295	−0.200	−0.240	−0.215	−0.205
NI	−0.231	−0.249	−0.259	−0.212	−0.218	−0.163*	−0.166*
N	−0.085*	−0.175*	−0.125*	−0.049*	−0.077*	−0.026*	−0.012*
NS	−0.300	−0.332	−0.334	−0.297	−0.270	−0.242	−0.240
Global average	−0.347	−0.402	−0.399	−0.340	−0.369	−0.328	−0.343

The correlations showed above were statistically significant at the level of 0.05, except for those marked*. cpRNFL, circumpapillary retinal nerve fiber layer; TS, superotemporal; T, temporal; TI, inferotemporal; NI, inferonasal; N, nasal; NS, superonasal.

## Discussion

Glaucoma has gradually become known as a condition that has macular involvement in the early stage even with well-preserved VA (6, 7, 21), and this macular damage greatly affects vision-related quality of life among glaucoma patients (10, 22). Various visual function tests have been studied for the early detection of macular damage, such as VF tests (23), CS tests (11), and letter recognition tasks (24). Although these tests are workable for glaucoma discrimination, the VA test is still the most convenient and simplest test to apply in clinical practice. However, there have been limited studies regarding VA in glaucoma patients.

Shah et al. confirmed that in foveal viewing, while conventional letters are good stimuli for detecting defocus, high-pass filtered letters were less vulnerable to optical defocus and more sensitive to neural limitations in conditions such as AMD (14, 25). A significant difference between the detection and resolution thresholds of high-pass letters, owing to undersampling as a result of photoreceptor loss, may be responsible for the VA loss measured by the high-pass letter chart (14, 25). As in the case of glaucoma, undersampling resulting from RGC damage may also affect the resolution threshold of high-pass letters in the fovea condition. Moreover, as contrast-sensitive neurons, RGCs play an important role in detecting differences in contrast (24, 26). Previous studies have shown that the low-contrast letter test could detect visual loss in patients with ocular hypertension and glaucoma, even when conventional VA was normal (17). Kwon et al. also pointed out an elevated contrast requirement for letter recognition in central vision (24).

In the present study, we sought to measure the effect of both a high-pass design and a low-contrast setting on the pattern resolution of glaucomatous eyes under foveal viewing. We wanted to first confirm whether low-contrast high-pass optotypes could better serve as stimuli for glaucoma detection and then to assess the structure-function relationships between retinal thicknesses measured by SD-OCT and VAs to find the potential structural basis that undermined central pattern vision in glaucomatous eyes.

In agreement with previous studies (14, 25), the recognition thresholds for high-pass optotypes were significantly higher than those for conventional letters in fovea viewing. As the contrast decreased, even higher thresholds were shown (Figure 4). Given that most of the low-frequency information was extracted from the stimuli, increasing letter size was obliged to turn the higher spatial frequencies into lower spatial frequencies so that the visual system could resolve the content. When the low contrast setting was superimposed, an even larger size was required. However, compared with healthy control eyes, glaucomatous eyes showed a greater level of disagreement between conventional VA and high-pass VA (Figure 5). Even at the 100% contrast level, there is a significant difference between the two VAs. Part of this might be explained by the slightly lopsided VA level between the glaucoma patients with normal vision and the healthy controls (−0.09 ± 0.08 logMAR vs. −0.17 ± 0.07 logMAR). However, the peculiar property of high-pass design may also account for this. We also notice that the lower the contrast was, the larger this difference was. Among glaucoma patients, conventional VA measurements were nearly 3 lines (0.28 logMAR) better than 100% high-pass acuity measurements compared with a figure of 2 lines (0.22 logMAR) in the healthy control group. For the data between the 1.25% high-pass VA and conventional VA, the disparity enlarged to 11 lines (1.08 logMAR) vs. 9 lines (0.93 logMAR). However, while the findings of low-contrast high-pass VA charts are potentially clinical meaningful, their value will be greatly diminished if large test-retest variabilities (TRVs) exists. Figure 3 graphically displays the results of repeated measurements for each VA chart among glaucoma patients with normal vision and healthy controls separately. We found that TRV for each VA chart were similar in both groups. In line with previous studies, the TRV for the high-pass VA chart at 100% contrast setting are lower than that for the conventional VA chart (27). Poorer TRVs were showed in glaucoma patients both for high-pass VA charts and conventional VA chart. TRVs for high-pass VA charts with lower contrast settings were even higher (TRV values varying from ± 0.10 to ± 0.14 logMAR). However, here TRVs were measured only in 20 glaucoma patients and 20 normal subjects with these charts. Future work is require to explore this issue in a larger sample size. We can also see from Figure 5 that the significant regression slopes indicated that the difference between conventional VA and high-pass VA was greater in glaucoma subjects with worse acuity. This suggests that the high-pass charts are able to detect functional loss as a result of glaucoma damage when conventional VA is still normal. In addition, 1.25% low-contrast high-pass VA had the highest sensitivity and specificity of these techniques.

Kim et al. pointed out that only weak structure-function relationships were shown between macular mGCC parameters and conventional VA (*r* = −0.363 to −0.410), and the global average cpRNFL thickness showed the highest correlation with coefficient value of −0.447 (28). Our findings showed that most of the RGC-related parameters from macular SD-OCT scans correlated better with high-pass VAs, particularly the 1.25% low-contrast high-pass VAs. The 1.25% low-contrast high-pass VA showed stronger structure-function correlations with nasal-side RGC-related thickness than conventional VA with statistical significance. However, there were only weak-to-moderate correlations between cpRNFL and VAs. These results are different from those in the study by Kim et al., which may be due to the different spectrum of glaucomatous damage involved. Here, we focused on the population with relatively good VA (BCVA equal to or better than 0.20 logMAR on ETDRS chart) but no requirement for VF defects. Given that the RNFL contains not merely nerve fibers but also non-neural or glial tissues, it is readily comprehensible that macular thickness parameters, especially RGC-related ones, are supposed to demonstrate stronger structure-function relationships than cpRNFL parameters with functional parameters that are sensitive to glaucoma damage.

We acknowledge that our study has some major limitations. First, our study is failed to make comparisons between conventional chart and high-pass chart at equal contrast levels. We hold the view that it is better to have a standard reference, like ETDRS chart used in the current study, for the multi-contrast comparisons. However, these have already been integrated within the scope of our further study. Second, as one of the main purposes of this study was to investigate whether low-contrast high-pass acuity charts were able to detect functional loss as a result of glaucoma damage when conventional VA was still quite good, we only included glaucoma patients with ETDRS logMAR VA equal to or better than 0.20 logMAR, which does not cover the full spectrum of glaucomatous damage. Third, no longitudinal investigations were conducted to determine RGC damage and high-pass VA loss over time, which prevents the study from indicating the ability of high-pass stimuli to detect glaucomatous progression. Fourth, the participants included were relatively young generally, therefore the findings are not applicable for the older population with glaucoma in whom cataract and macular degeneration are common.

To summarize, VA measurements taken with low-contrast high-pass acuity charts appear to be more sensitive in detecting central visual loss in glaucoma than those taken with conventional charts. Nasal-side macular GCL thinning appears to be associated with low-contrast high-pass visual loss in glaucomatous eyes.

## Data Availability Statement

The raw data supporting the conclusions of this article will be made available by the authors, without undue reservation.

## Ethics Statement

The studies involving human participants were reviewed and approved by the ethics committee of the Zhongshan Ophthalmic Center (NO.2019KYPJ115). The patients/participants provided their written informed consent to participate in this study.

## Author Contributions

YW, ZC, and MY conceived and designed the study. ZC, YK, and HC conducted the coding and programming. YW, ZC, CZ, YY, JX, and MY analyzed and interpreted the patient data. YW analyzed the data and was a major contributor in writing the manuscript. All authors provided critical appraisal and final approval of the manuscript.

## Conflict of Interest

The authors declare that the research was conducted in the absence of any commercial or financial relationships that could be construed as a potential conflict of interest.
